# Bruton Tyrosine Kinase Inhibitor Ibrutinib and Pericardial Tamponade: A Case Report

**DOI:** 10.7759/cureus.101341

**Published:** 2026-01-12

**Authors:** Mariana Baptista, Maria Soares, Rosélia Lima, Cristina Ferreira, Tiago Gregório

**Affiliations:** 1 Internal Medicine, Unidade Local de Saúde de Gaia e Espinho, Vila Nova de Gaia, PRT; 2 Hematology, Unidade Local de Saúde de Gaia e Espinho, Vila Nova de Gaia, PRT

**Keywords:** adverse drug reaction, cardiac tamponade, cardiotoxicity, chronic lymphocytic leukemia, ibrutinib, pericardial effusion

## Abstract

Ibrutinib is a Bruton’s tyrosine kinase (BTK) inhibitor widely used in the treatment of B-cell malignancies, including chronic lymphocytic leukemia (CLL). While effective, it is associated with cardiovascular adverse events, most commonly atrial fibrillation (AF) and hypertension. Pericardial complications, such as pericardial effusion and cardiac tamponade, are extremely rare.

We report the case of a 66-year-old woman with CLL treated with ibrutinib who presented with progressive dyspnea, signs of peripheral hypoperfusion, and a new-onset episode of AF. Point-of-care echocardiography revealed a large pericardial effusion with features of cardiac tamponade, requiring urgent pericardiocentesis, with hemodynamic stabilization. Ibrutinib was discontinued due to clinical concern for a possible drug-related serosal reaction, followed by complete resolution of pericardial and pleural effusions and sustained clinical improvement.

This case highlights a rare but potentially life-threatening complication that may be associated with ibrutinib therapy. Clinicians should maintain a high index of suspicion for cardiac tamponade in patients receiving BTK inhibitors who present with nonspecific cardiopulmonary symptoms. Prompt echocardiographic evaluation and individualized management decisions, including drug discontinuation, are key to achieving favorable outcomes.

## Introduction

Ibrutinib is an oral Bruton’s tyrosine kinase (BTK) inhibitor approved by the U.S. Food and Drug Administration (FDA) in 2013 for the treatment of several B-cell malignancies, including chronic lymphocytic leukemia (CLL), mantle cell lymphoma, Waldenström’s macroglobulinemia, marginal zone lymphoma, and chronic graft-versus-host disease. It irreversibly inhibits BTK, a key component of B-cell receptor signaling, thereby suppressing malignant B-cell proliferation and survival [1]. As a first-line treatment for CLL, ibrutinib has demonstrated significant improvements in overall and progression-free survival [2].

Despite its clinical benefits, ibrutinib is associated with a well-documented spectrum of adverse effects. Common toxicities include diarrhea, fatigue, and neutropenia [3]. Cardiovascular complications are particularly noteworthy and require close monitoring. Atrial fibrillation (AF) is the most frequent cardiac event, with reported rates ranging from 3.3 per 100 person-years in clinical trials to up to 16% in real-world settings. Hypertension is also common, occurring in as many as 80% of patients [4,5]. Less frequently, serosal inflammatory events such as pericardial and pleural effusions have been reported. According to pharmacovigilance data from the U.S. Food and Drug Administration Adverse Event Reporting System (FAERS) database, pericardial effusion has been reported in approximately 1% of ibrutinib-related adverse event (AE) reports, while cardiac tamponade accounts for fewer than 0.25% and pericarditis for fewer than 0.5% of reported cases [6]. Although uncommon, these complications can be potentially life-threatening, as rapid accumulation of pericardial fluid may impair ventricular filling, reduce cardiac output, and precipitate obstructive shock if not promptly recognized and emergently treated, often necessitating drug discontinuation [3,4].

Beyond its intended inhibition of BTK, ibrutinib’s cardiovascular toxicity is largely attributed to off-target effects on kinases involved in cardiac and vascular homeostasis, which can disrupt normal electrophysiology and myocardial remodeling, thereby increasing the risk of arrhythmias, hypertension, heart failure, and, more rarely, serosal inflammation. Importantly, in patients with CLL, pericardial and pleural effusions may arise from multiple mechanisms, including malignant involvement, infection, inflammatory processes, or drug-related toxicity, highlighting the need for careful clinical assessment.

Accordingly, comprehensive baseline cardiovascular evaluation, including blood pressure measurement, electrocardiography, and echocardiography when indicated, is recommended for patients initiating or receiving ibrutinib, with subsequent investigations guided by new or worsening cardiopulmonary symptoms [7,8].

This case report presents a rare instance of cardiac tamponade occurring in temporal association with ibrutinib therapy for CLL. It highlights the importance of early recognition and management of serious cardiovascular events in patients receiving BTK inhibitors, especially as their use becomes increasingly widespread in hematologic oncology.

## Case presentation

We report the case of a 66-year-old female patient with a Clinical Frailty Scale score of 4, a history of depressive disorder and osteoporosis, and a diagnosis of high-risk B-cell chronic lymphocytic leukemia (CLL-B) classified as Binet stage C, Rai stage IV, with deletion of the long arm of chromosome 13 [del(13q)], deletion of the short arm of chromosome 17 [del(17p)], and unmutated immunoglobulin heavy-chain variable region (IgHV) genes, findings associated with an adverse prognosis. She was receiving first-line treatment with ibrutinib.

Prior to initiating therapy, a baseline electrocardiogram and chest radiograph were unremarkable. A transthoracic echocardiogram was not performed, according to our institution’s protocol, which aligns with contemporary cardio-oncology guidelines and reflects a structured, risk-anchored assessment.

**Figure 1:**
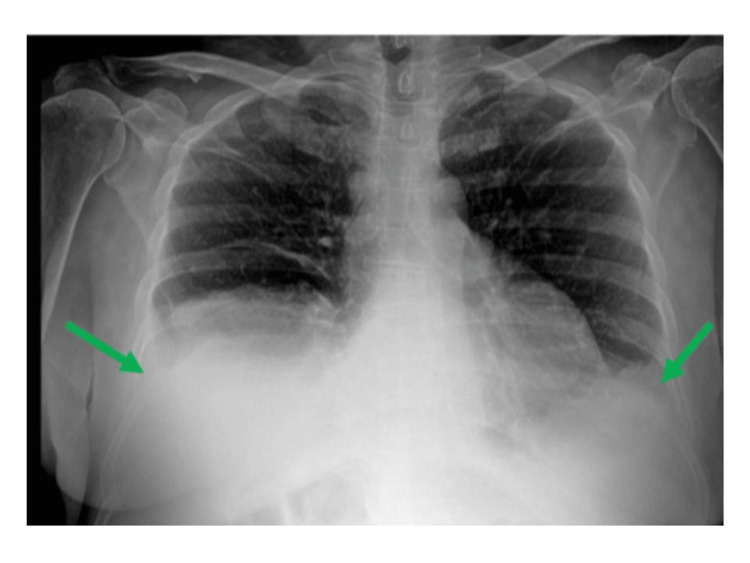
Chest radiograph demonstrating bilateral pleural effusion two months after the initiation of ibrutinib therapy

Two months after starting ibrutinib, she developed new-onset dyspnea. A repeat chest radiograph revealed a small-to-moderate bilateral pleural effusion (Figure 1), and an echocardiogram showed a small pericardial effusion adjacent to the right atrium, mild tricuspid regurgitation, no chamber compression, and preserved biventricular function. Thoracentesis revealed transudative pleural fluid. Flow cytometry identified a small population of malignant B cells, but given the presence of blood, low cellularity, absence of neoplastic cells on cytology, and lack of exudative features, these findings were attributed to peripheral blood contamination. As there was no evidence of disease progression, ibrutinib was continued, and diuretic therapy with furosemide 40 mg once daily was initiated.

**Figure 2:**
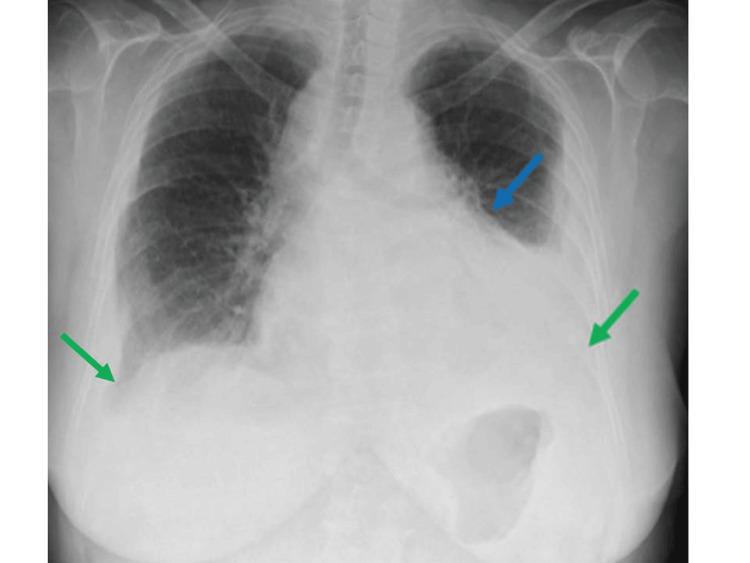
Chest radiograph showing an enlarged cardiac silhouette (blue arrow) and bilateral pleural effusion (green arrows)

Eight months later, the patient presented to the Emergency Department (ED) with a five-day history of dyspnea on mild exertion and generalized malaise. On admission, she was afebrile, with a blood pressure of 128/65 mmHg and a normal sinus rhythm. Despite a seemingly normal blood pressure, clinical examination revealed signs of poor perfusion, including livedo reticularis in the extremities and prolonged capillary refill time. Additional findings included jugular venous distention, muffled heart sounds, tachypnea at 28 breaths per minute, and decreased breath sounds in the mid-zone of the left lung. Initial laboratory studies showed hyperlactatemia of 3.4 mmol/L (0-2 mmol/L), supporting the hypothesis of poor tissue perfusion, an elevated C-reactive protein (CRP) of 28 mg/dL (0-0.5 mg/dL) and a mildly elevated erythrocyte sedimentation rate of 27 mm/h (< 20mm/h), without leukocytosis or neutrophilia. A chest X-ray demonstrated an enlarged cardiac silhouette and moderate bilateral pleural effusion (Figure 2).

**Figure 3:**
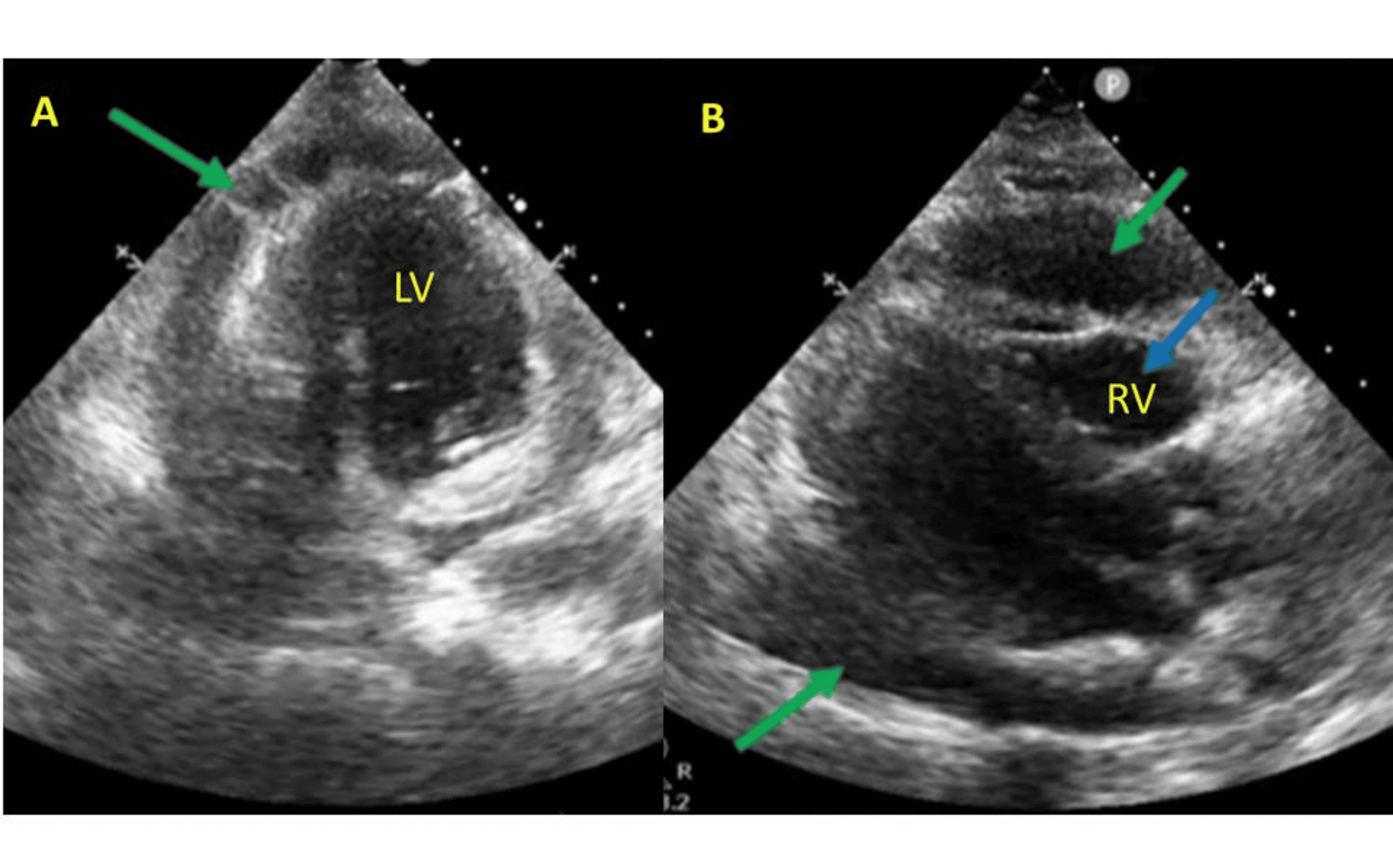
Point-of-care echocardiographic findings of cardiac tamponade at presentation A: Apical four-chamber point-of-care echocardiographic view obtained at Emergency Department admission, demonstrating a large pericardial effusion (green arrow). B: Parasternal long-axis point-of-care echocardiographic view showing a large pericardial effusion (green arrows) with echocardiographic features of cardiac tamponade, including diastolic right ventricular collapse (blue arrow). These images were acquired during hemodynamic instability; formal Doppler and M-mode analysis were not performed at this stage. LV, left ventricle; RV, right ventricle

While in the ED, the patient developed an episode of AF with rapid ventricular response (185 beats per minute), accompanied by more hemodynamic instability - marked hypotension (60/45 mmHg), worsening signs of peripheral hypoperfusion, and a significant increase in serum lactate to 6.4 mmol/L (0-2 mmol/L). The arrhythmia was self-limited. At that time, a point-of-care echocardiogram revealed a large pericardial effusion with echocardiographic features consistent with cardiac tamponade, including diastolic collapse of the right ventricle, systolic collapse of the right atrium, a plethoric inferior vena cava with reduced respiratory variation, and the presence of the "swinging-heart" sign (Figures 3A, 3B). Visual assessment (“eyeballing”) also suggested impaired left ventricular systolic function, likely at least moderate; however, formal quantification of ejection fraction and strain analysis were not performed at that stage.

Although ultrasound confirmed tamponade, the transthoracic acoustic window was documented as incompatible with safe echocardiography-guided pericardiocentesis. As the patient had stabilized, a computed tomography (CT) scan was obtained, confirming a large pericardial effusion measuring 40 mm with imaging signs of tamponade. CT-guided pericardiocentesis was therefore performed emergently, yielding 650 mL of serohematic, non-purulent fluid, and a pericardial drain was left in situ. Given the emergent nature of the procedure, a full diagnostic pericardial fluid workup (biochemistry, cytology, microbiology, ADA, and viral PCR) was not performed. A sample collected via the drain for flow cytometry (immunophenotyping) could not be processed due to technical limitations.

Concomitantly, treatment with colchicine 0.5 mg twice daily was initiated, with a planned duration of three months, as inflammatory involvement of the pericardium could not be excluded. Due to delayed follow-up, the patient ultimately completed six months of therapy. Non-steroidal anti-inflammatory drugs (NSAIDs) were avoided due to transient hemodynamic instability, tissue hypoperfusion, and a recent serohematic effusion. Given the clinical concern for a possible ibrutinib-related adverse reaction, ibrutinib was discontinued.

The patient experienced a transient episode of AF, with a CHA₂DS₂-VA score of 1 based on age, warranting consideration of anticoagulation. However, anticoagulation was withheld due to the self-limited nature of the arrhythmia, the immediate post-procedural bleeding risk following pericardiocentesis in the setting of a serohematic effusion, and discontinuation of the suspected causal agent (ibrutinib). As sinus rhythm was maintained after hemodynamic stabilization and no recurrent arrhythmia occurred, beta-blocker therapy was not initiated during the index hospitalization.

The drain was clamped the following day, with a residual output of 70 mL, and was later removed without complications.

A transthoracic echocardiogram performed during the index hospitalization, after pericardiocentesis, showed left ventricular systolic function at the lower limit of normal, with an ejection fraction of 50% by the biplane Simpson method and a reduced global longitudinal strain (−11.2%), suggesting subclinical left ventricular systolic dysfunction. Right ventricular function was preserved (TAPSE 16 mm; tricuspid S′ 9.9 cm/s), and only minimal residual pericardial effusion was present. A low dose of ramipril was initiated as prognostic therapy in view of the impaired strain parameters.

The patient subsequently showed a favorable clinical course, with negative blood cultures, complete resolution of pleural effusions and near-complete resolution of the pericardial effusion, along with normalization of inflammatory markers within one week of hospitalization and by the time of discharge.

Five months after discontinuing ibrutinib, laboratory evidence of CLL-B progression led to the initiation of venetoclax, subsequently combined with six cycles of rituximab. Although venetoclax is not associated with routine cardiotoxicity requiring systematic surveillance, the patient’s recent history of cardiac tamponade and left ventricular systolic dysfunction warranted individualized cardiac assessment and a low threshold for echocardiographic re-evaluation, rather than scheduled serial imaging. The patient remained clinically stable during therapy.

At the six-month outpatient Cardiology follow-up, she remained clinically stable without recurrence of serosal involvement. Thoracic CT confirmed the absence of pleural or pericardial effusion. Transthoracic echocardiography showed a mildly reduced left-ventricular ejection fraction (45%), with a normal-appearing pericardium without effusion. Guideline-directed medical therapy was optimized with the addition of bisoprolol and dapagliflozin, in addition to the previously initiated ramipril. At this stage, the need for long-term anticoagulation was reassessed. Given the paroxysmal and transient nature of the AF episode, its temporal association with ibrutinib exposure, the absence of recurrence after drug discontinuation, and the presence of thrombocytopenia and anemia related to CLL progression, anticoagulation was not initiated due to an unfavorable bleeding risk profile.

One year later, a follow-up echocardiogram demonstrated recovery of left-ventricular systolic function, with a left ventricular ejection fraction of 55% and no evidence of pericardial effusion or other relevant abnormalities. A summary of the patient’s clinical course is presented in Table 1.

**Table 1:** Clinical timeline of events from diagnosis of high-risk B-cell chronic lymphocytic leukaemia and initiation of first-line ibrutinib therapy ECG, electrocardiogram; CLL-B, B-cell chronic lymphocytic leukemia; CRP, C-reactive protein; CT, computed tomography; LV, left ventricle; LVEF, left ventricular ejection fraction; GLS, global longitudinal strain; GDMT, guideline-directed medical therapy.

Time point	Clinical findings	Management
Baseline (pre-ibrutinib)	Normal ECG and chest radiograph. No cardiovascular history or symptoms. Baseline echocardiography not performed per the institutional cardio-oncology protocol.	Ibrutinib initiated as first-line therapy for high-risk CLL-B.
Two months	New-onset dyspnea. Chest X-ray: small-to-moderate bilateral pleural effusion. Echocardiogram: small pericardial effusion adjacent to the right atrium, preserved biventricular function. Thoracentesis: transudative pleural fluid.	Ibrutinib continued. Furosemide 40 mg daily initiated.
Eight months	Five-day history of progressive dyspnea and malaise. Clinical signs of hypoperfusion. CRP elevated (28 mg/dL). Chest X-ray: enlarged cardiac silhouette and bilateral pleural effusion.	Emergency Department admission.
Emergency Department presentation	Transient atrial fibrillation with rapid ventricular response and hypotension. Point-of-care echocardiography: large pericardial effusion with tamponade physiology, visual estimation suggested impaired LV systolic function.	Hemodynamic stabilization. Urgent CT performed.
Same day	CT scan: large pericardial effusion (40 mm) with signs of tamponade.	CT-guided pericardiocentesis performed; 650 mL serohematic fluid drained; pericardial drain placed.
Hospitalization	Limited pericardial fluid analysis due to emergent setting. Post-procedure echocardiogram: LVEF 50%, GLS −11.2%, minimal residual pericardial effusion.	Colchicine initiated (planned three months). Ibrutinib discontinued. A low dose of ramipril started. No anticoagulation initiated.
Discharge (≈ 1 week)	Resolution of pleural effusions, near-complete resolution of pericardial effusion. Normalization of inflammatory markers.	Clinical stability at discharge.
Five months post-discontinuation	Laboratory evidence of CLL-B progression.	Venetoclax initiated and later combined with rituximab. Continued cardiology follow-up.
Six-month cardiology follow-up	No recurrence of serosal effusions. Echocardiography: LVEF 45%.	GDMT optimized (bisoprolol, dapagliflozin added). Anticoagulation reconsidered but withheld.
One-year follow-up	Echocardiography: recovery of LV systolic function (LVEF 55%) and no pericardial effusion.	Sustained clinical and echocardiographic improvement.

## Discussion

Ibrutinib has significantly advanced the treatment of B-cell malignancies, including CLL-B [2]. While its efficacy is well established, accumulating evidence has identified a spectrum of cardiovascular AEs, most commonly atrial fibrillation and hypertension, and more rarely, pleural and pericardial effusions complicated by cardiac tamponade [9].

Cardiac tamponade as a complication of ibrutinib therapy remains exceedingly uncommon, with few cases reported in the literature. Barton et al. described a 57-year-old woman with CLL-B who developed non-malignant hemorrhagic pericardial effusion leading to cardiac tamponade after prolonged ibrutinib therapy, requiring pericardiocentesis and drug discontinuation [10]. Similarly, Candır et al. reported a case of non-hemorrhagic pericardial effusion progressing to tamponade in a 64-year-old man receiving ibrutinib, without evidence of infection or malignancy, suggesting drug-induced serositis [11]. Our case mirrors these reports, particularly regarding the delayed onset of serosal involvement and complete resolution after ibrutinib withdrawal, strengthening the plausibility of a drug-related mechanism.

The pathophysiology underlying ibrutinib-associated pericardial complications remains incompletely understood. Proposed mechanisms include off-target effects, kinase inhibition, resulting in platelet dysfunction, increased vascular permeability, and immune-mediated serosal inflammation. Such effects may predispose susceptible patients to hemorrhagic or inflammatory effusions, potentially progressing to tamponade in the absence of early recognition [9].

In the present case, a clear temporal relationship was observed between ibrutinib exposure and progressive development of pleural and pericardial effusions, culminating in hemodynamic compromise and echocardiographically confirmed cardiac tamponade (with right atrial and right ventricular collapse, a dilated inferior vena cava with reduced respiratory variation, and the "swinging heart" sign [12,13]). Following urgent pericardiocentesis and subsequent discontinuation of ibrutinib, both pericardial and pleural effusions resolved completely and did not recur, further supporting a causal association.

However, the emergent clinical context precluded comprehensive pericardial fluid analysis, and technical limitations prevented successful immunophenotyping. Consequently, malignant, infectious, and inflammatory aetiologies cannot be definitively excluded, representing a major diagnostic limitation. Accordingly, the association between ibrutinib and serosal involvement in this case is best framed as possible-probable rather than definitive. The main alternative aetiologies considered for the pericardial and pleural effusions are summarized in Table 2.

**Table 2:** Clinical, laboratory, and imaging findings supporting and arguing against each suspected aetiology CLL-B, B-cell chronic lymphocytic leukemia.

Suspected aetiology	Findings supporting this diagnosis	Findings arguing against this diagnosis
Ibrutinib-related serosal toxicity	Clear temporal relationship with ibrutinib exposure; Progressive pleural and pericardial effusions during therapy; Complete resolution of effusions after drug discontinuation; Similar cases reported in the literature.	Lack of comprehensive pericardial fluid analysis limits definitive exclusion of other causes.
Malignant serositis (CLL-B-related)	Underlying high-risk CLL-B (Binet C, Rai IV); Prior pleural effusion with detection of a small B-cell population on flow cytometry.	Pleural fluid was transudative; Low cellularity and probably blood contamination of the pleural sample; Absence of malignant cells on cytology of the pleural sample; No clinical evidence of CLL-B progression at the time of tamponade; Complete and sustained resolution of serosal effusions after pericardiocentesis and ibrutinib discontinuation, despite subsequent laboratory evidence of CLL-B progression.
Infectious pericarditis/serositis	Elevated inflammatory markers; Large pericardial effusion with tamponade physiology.	Afebrile presentation and absence of typical pericarditis features (no chest pain, no pericardial rub, no diagnostic ECG changes); Did not fulfil formal diagnostic criteria for acute pericarditis; No leukocytosis or neutrophilia; Negative blood cultures; No epidemiological risk factors or clinical focus of infection; Non-purulent pericardial fluid.
Inflammatory pericardial involvement (non-infectious)	Elevated inflammatory markers; Large pericardial effusion with hemodynamic compromise; Favorable response to colchicine therapy.	Absence of typical pericarditis features (no chest pain, no pericardial rub, no diagnostic ECG changes); Did not fulfil formal diagnostic criteria for acute pericarditis; Only mildly elevated erythrocyte sedimentation rate.

To support causality assessment, we applied the Naranjo Adverse Drug Reaction Probability Scale, which classifies adverse drug reactions as definite (≥9), probable (5-8), possible (1-4), or doubtful (≤0) based on a cumulative score. Although widely used, the scale should be interpreted cautiously in complex clinical scenarios, such as oncology and targeted therapies. In this case, the patient’s score of 5 categorized the reaction as “probable” [14]. Importantly, this score should be viewed as supportive rather than confirmatory evidence, particularly in the absence of complete diagnostic data. 

Although the patient did not fulfil formal diagnostic criteria for acute pericarditis, inflammatory involvement of the pericardium could not be safely excluded, given the presence of a large pericardial effusion, elevated inflammatory markers, and the absence of an alternative definitive etiology. Current guidelines do not provide strong evidence to support routine colchicine use in patients with pericardial effusion or tamponade in the absence of diagnostic criteria for acute pericarditis. However, in the context of a severe clinical presentation and diagnostic uncertainty, initiation of colchicine was based on an individualized risk-benefit assessment and implemented with close monitoring for adverse effects [15]. In our case, although a three-month course was planned, the patient completed a longer course due to delays in follow-up rather than clinical indication. NSAIDs are commonly included in first-line therapy, but they may be unsuitable in certain clinical contexts such as hemodynamic compromise or impaired perfusion, which was the case in our patient. Colchicine monotherapy was considered therefore a reasonable guideline-aligned approach.

The favorable clinical course, with sustained resolution of serosal effusions following drug discontinuation, aligns with previously reported cases where ibrutinib was implicated in serosal inflammation leading to effusions, which resolved upon cessation of the drug [10,11,16]. This reinforces the importance of considering drug-related toxicity in the differential diagnosis of unexplained pericardial or pleural effusions in patients receiving BTK inhibitors.

Management of ibrutinib-associated pericardial complications requires a high index of suspicion, especially in patients presenting with nonspecific cardiopulmonary symptoms such as dyspnea and fatigue. Early recognition through imaging modalities, particularly echocardiography, is essential. Therapeutic strategies may include pericardiocentesis and anti-inflammatory therapy, as in our patient. In selected cases of recurrent, loculated, or refractory effusions, prolonged pericardial drainage or surgical interventions such as pericardial window creation or pericardiectomy may also be required.

Decisions regarding the continuation or cessation of ibrutinib must be individualized, balancing oncologic benefit against the risks of recurrent cardiovascular complications [10].

Contemporary cardio-oncology recommendations, including guidance from the National Comprehensive Cancer Network (NCCN) and expert consensus statements, emphasize the importance of comprehensive cardiovascular assessment before and during BTK inhibitor therapy such as ibrutinib. Baseline evaluation should include a thorough cardiac history, ECG, blood pressure measurement, routine laboratory tests and echocardiography when indicated, with subsequent surveillance guided by clinical evolution [5-7]. This proactive approach facilitates early identification and management of treatment-related toxicities. In retrospect, in our case, earlier consideration of possible ibrutinib-related adverse effects and the safety of maintaining therapy might have helped prevent progression to a life-threatening complication. Nonetheless, this decision is often complex, given the drug’s substantial therapeutic benefit.

In summary, this case underscores the importance of vigilance for rare but potentially life-threatening cardiovascular AEs possibly associated with ibrutinib. Clinicians should maintain a low threshold for cardiac evaluation in patients symptomatic and receiving BTK inhibitors and adopt individualized management strategies. Further research is warranted to clarify the mechanisms, risk factors, and optimal prevention and management of cardiovascular toxicities related to BTK inhibition, to develop strategies for risk mitigation.

## Conclusions

This case report describes a rare but clinically significant presentation of possible-probable ibrutinib-related serositis complicated by cardiac tamponade in a patient with CLL-B. The temporal relationship with drug exposure and complete resolution after discontinuation supports a drug-related mechanism. However, the absence of comprehensive pericardial fluid analysis precludes definitive exclusion of malignant or inflammatory/infectious aetiologies. This case underscores the importance of maintaining a high index of suspicion for severe cardiovascular complications in patients receiving BTK inhibitors. A low threshold for cardiac imaging in symptomatic patients and individualized, multidisciplinary management strategies are essential to balance oncologic benefit with cardiovascular safety. Further research is warranted to better define the mechanisms, risk factors, and optimal management of cardiovascular toxicities associated with BTK inhibitors.
